# Effect of the time interval between cataract surgery for both eyes on mental health outcome: a cohort study of 585,422 patients

**DOI:** 10.1186/s12886-021-01876-9

**Published:** 2021-03-01

**Authors:** Chiun-Ho Hou, Ken-Jen Chen, Jiahn-Shing Lee, Ken-Kuo Lin, Christy Pu

**Affiliations:** 1 Department of Ophthalmology, Chang Gung Memorial Hospital, Linkou Medical Center, Taoyuan, Taiwan; 2 Institute of Public Health, School of Medicine, National Yang Ming Chiao Tung University, 155 Linong Street. Sec 2, Peitou, Taipei, Taiwan; 3 Department of Ophthalmology, Change Gung Memorial Hospital, Xiamen, People’s Republic of China; 4 Department of Medicine, College of Medicine, Chang Gung University, Taoyuan City, Taiwan

**Keywords:** Cataract surgery, Interval, Mental health, Universal health coverage

## Abstract

**Background:**

Cataract surgeries can improve mental health outcomes. However, previous studies have not investigated whether the time interval between cataract surgeries for 2 eyes affects mental health outcomes.

**Methods:**

We used the whole-population National Health Insurance (NHI) claims data from Taiwan to conduct a cohort study. Patients who received cataract surgeries for both eyes were identified (*n* = 585,422). The mental health inpatient and outpatient consultations received by these patients were analyzed, with different time intervals (< 3, 3 to 6, 6 to 12, and > 12 months) between the surgeries. Negative binominal regression was performed to estimate the interaction of the first eye surgery with the time interval.

**Results:**

The number of mental health consultations was lowest among patients with a time interval of < 3 months (1.783–1.743, *P* < .001), and a negative dose response effect was observed, such that a longer time interval corresponded to a lower reduction in the number of mental health consultations. For patients with a time interval of > 12 months, the predicted number of mental health consultations increased from 1.674 to 1.796 (*P* < .001).

**Conclusions:**

Given a patient expected to receive surgeries for both eyes within 1 year, scheduling both surgeries within a short time interval may be beneficial for maximizing the effects of cataract surgery in reducing the number of mental health consultations.

## Introduction

Visual loss has been reported to be associated with depression and an increased risk of suicide [1–3]. Cataracts, the most common treatable cause of visual impairment and blindness worldwide, have been reported to be associated with depression and anxiety [4–6]. One study showed that patients with poorer visual acuity awaiting cataract surgery were more likely to be depressed than those with better visual acuity [7]. Surgery can effectively treat cataracts, and according to some studies, cataract surgery may be an effective strategy for ameliorating depression and anxiety among people with cataracts. However, the results from previous studies are inconsistent.

In their study of 102 patients undergoing phacoemulsification and intraocular lens implantation for bilateral cataracts, Ishii, Kabata, and Oshika found that changes in depression measured using the Beck Depression Inventory were significantly correlated with changes in vision-related quality-of life after surgery [8]. Similar positive effects of cataract surgeries for ameliorating depression or psychological distress have been reported by other studies [9–11]. However, not all studies have confirmed such positive effects of cataract surgery on depression [12, 13]. Anxiety and depression may be increased after surgery in patients whose vision does not improve [10, 14].

Most studies investigating the effects of cataract surgery on mental health or subsequent health status have relied on small sample sizes or patients with specific characteristics. In addition, few studies have used substantial population-representative data. For example, Harwood provided sound evidence from a randomized controlled trial (RCT) of the effect of cataract surgery on anxiety and depression in the UK [15]; however, they included only women aged over 70 years. An Australian-based RCT conducted with 45 adult participants (mean age = 73.7 years) reported that patients with improved vision after surgery had improved mood compared with patients without vision improvements, who had unchanged or worse depression [14]. A cohort study with 122 adult participants receiving cataract surgery and 92 not receiving surgery found that cataract surgery had no association with mobility or the occurrence of falls [16].

In addition to providing further evidence of the effects of first-eye cataract surgery on mental health, this study aims to address two gaps in the literature. First, few studies have examined the effect of second-eye cataract surgery on depression and psychological distress. Second-eye surgeries represent a substantial proportion of the total number of cataract surgeries [17]. One-sided cataracts can cause functional problems such as disturbed motion perception and disturbed stereo acuity, [18, 19] and the benefits of second-eye cataract surgery in terms of self-reported visual symptoms and function have been well documented [20]. Despite the visual benefits of second-eye surgery, however, secondary patient outcomes such as reduction in depression and physiological distress have rarely been investigated. The effect of cataract surgery on objective outcomes such as health service utilization for depression and anxiety have rarely been studied at the population level. Among the limited studies, Meuleners and Hendrie used administrative data and reported an 18.8% reduction in the number of mental health consultations for depression and anxiety within the year following first-eye cataract surgery [5].

The effect of the time interval between first- and second-eye surgeries on patient outcomes has yet to be investigated. When medical resources are insufficient for meeting the demand for cataract surgery, second-eye procedures are often considered secondary, and this may lead to long waiting times. Long time intervals between the two surgeries may lead to low patient perceived value of the surgeries, and this is especially true for patients with a short remaining survival duration [21].

The objectives of this study are (1) to determine the effect of cataract surgery on medical utilization for depression and anxiety for both first- and second-eye surgeries, and (2) to determine whether second eye surgery receipt and the time interval between cataract surgeries affects utilization outcomes for depression and anxiety treatment.

## Methods

This retrospective cohort study had a study period from 2007 to 2017. We used all-population claims data (for approximately 23 million individuals) from the Taiwan National Health Insurance (NHI) database. Enrollment in the NHI is compulsory for all citizens. The NHI database is managed and distributed by the Ministry of Health, Taiwan. The NHI claims data contain all information on inpatient and outpatient health care utilization, including all medical care, prescriptions, and medical procedures received under the NHI. All individuals are included in the database, even if they do not have any medical claims in a given year. This study was approved by the Institutional Review Board (IRB) of National Yang-Ming University, which is a research review board formed and operated by the above named university. All data were anonymized before being released to researchers. Individual informed consents were waived (IRB name: Continuity of care, healthcare seeking behavior and patient outcome: case of chronic diseases, mental illness, ophthalmology and otorhinolaryngology. IRB number: YM107047E-2).

### Study subjects

We first selected all patients who received cataract surgeries for both eyes during the period from 2007 to 2017 (*n* = 633,883). We included only patients who had received cataract surgeries for both eyes because if only one cataract surgery was received during the period, then we would be unable to confirm whether that surgery was for the first or second eye. We then excluded patients who had first-eye cataract surgery in 2007 (*n* = 47,629). This exclusion criterion was necessary because at least 1 year of data on preoperative utilization of care for depression or mental distress would be necessary for analysis. We then excluded patients with missing variables, leaving us with a final sample of 585,422 patients.

### Exposure and outcome variables

We identified cataract surgeries and time intervals between first- and second-eye surgeries using NHI-specific codes for cataract surgeries (86008C, 86007C, 86009C). The time intervals between the 2 surgeries were defined as < 3, 3 to 6, 6 to 12, and > 12 months. The time interval was treated as a categorical variable instead of a continuous variable to account for nonlinearity in the effects. Outpatient and inpatient visits for mental health consultations were defined as consultations for depression, anxiety, and sleep problems. These consultations were identified using International Classification of Diseases, 9th Revision, Clinical Modification (ICD-9-CM) and ICD-10 codes as follows: ICD-9-CM: 296.2*, 296.3*, 311.*, 300.0*, 307.4*, and 780.5*; and ICD10: F32.*, F33.*, F41.*, F51.*, and G47.*. We excluded mental conditions that are unlikely to be related to cataracts, such as adjustment disorder. The number of mental health consultations was calculated for 1 year before and 1 year after surgery for the first eye.

### Covariates

Age and sex are basic demographic variables and were included in the regression models. Although health coverage is universal in Taiwan, utilization may still depend on one’s socioeconomic status because there may be indirect costs associated with medical care (such as caregiver costs). Therefore, we included low-income status, defined according to the official Taiwan government, as a variable. Because mental health can also be affected by other health problems, we included the Charlson comorbidity index to capture health conditions. Health care utilization can be influenced by medical resource availability, which can vary by area; therefore, we included an area measure (urban, suburban, or rural) in the analysis.

### Statistical methods

We categorized study participants into those with and without mental health consultations before and after cataract surgery for the first eye. Statistical differences in the variables of interest were determined using the *t* test for continuous variables and the chi-square test for categorical variables. We used a negative binominal regression model since the number of mental health consultations is a count variable. In addition, negative binominal models allow more flexibility in the assumption of variance, and hence are more appropriate than the Poisson models. The model included the first-eye surgery and added an interaction term for the first-eye surgery and the time interval between first- and second-eye surgeries. The purpose of the interaction term was to determine whether the effects of first-eye surgery on mental health consultations vary according to time intervals between the 2 eyes. Estimates were adjusted for repeated measures at the individual level according to cluster variance. The coefficient estimated for the interaction effect for nonlinear models should not be interpreted directly. Thus, marginal effects were computed for each variable according to the full derivatives of the dependent variables with respect to the variables in the model.

### Sensitivity analysis

To test the robustness of our estimates, we performed several sensitivity analyses. First, we estimated a model included only the first-eye surgery date and computed changes in mental health consultations 1 year before and 1 year after the surgery. Second, we recategorized time intervals into 2 categories by using different cutoff points according to the median number of days. Although the results revealed a similar trend compared with the original categorization, the alternative categorization did not seem more suitable because the majority of the participants had a time interval of fewer than 3 months. In addition, broadening the categories would lead to a loss of information. Third, we tested the same model without the interaction term between first-eye surgery and time interval. Because there were many model specifications, we selected our final model according to the Akaike information criterion (AIC). A model with a lower AIC is considered more efficient with respect to explanatory power. We then estimated the current model using a zero-inflated negative binominal model. However, the results were similar to those of the current negative binominal model; therefore, we retained the negative binomial models in this study.

## Results

Table 1 presents patient characteristics against mental health consultations. The average age was 69.3 (standard deviation [SD] = 9.5) years. Women underwent more surgeries than men did, constituting 57% of the sample. A higher percentage of women had mental health consultations prior to the first-eye surgery than did men (28.5 and 19.6% for women and men, respectively). Among those who had mental health consultations prior to the first-eye surgery (144, 841 [24.7%), the average number of mental health visits 1 year before the surgery was 7.15 (SD = 6.86), and the average number of visits 1 year after surgery was 6.2 (SD = 7.2) The time interval between the first- and second-eye surgeries was > 3 months for the majority (44.4%) of the patients.

**Table 1:** Mental health care utilization 1 year before and after cataract surgery for the first eye

		All patients	Pre-cataract surgery			Post-cataract surgery		
			Had mental service consultation	Did not have mental healthconsultation		Had mental service consultation	Did not have mental health consultation	
	Number of patients	585,422	144,841	440,581		147,414	438,008	
Age	Mean (SD)	69.31 (9.50)	70.11 (8.79)	69.05 (9.71)	< 0.001	70.03 (8.79)	69.07 (9.72)	< 0.001
	Median (IQR)	70 (63–76)	71 (64–76)	70 (63–76)		71 (64–76)	70 (63–76)	
	20–35	1062	88	974	< 0.001	114	948	< 0.001
	35–50	15,372	2301	13,071		2397	12,975	
	50–65	153,580	34,255	119,325		35,056	118,524	
	> = 65	415,408	108,197	307,211		109,847	305,561	
	Mental service consultation							
	Pre-surgery	144,841	–	–		103,364	41,477	
	Post-surgery	147,414	103,364	44,050		–	–	
	Number of consultations							
Number of mental consultation	Pre-surgery	1.77 (4.60)	7.15 (6.86)	–		6.18 (7.26)	0.28 (1.33)	< 0.001
	Median (IQR)	0 (0–0)	5 (2–11)	–		4 (0–10)	0 (0–0)	
Number of mental consultation	Post-surgery	1.79 (4.60)	6.20 (7.21)	0.35 (1.59)	< 0.001	7.13 (6.78)	–	
	Median (IQR)	0 (0–1)	4 (0–10)	0 (0–0)		5 (2–11)	–	
Sex	Male	249,637	49,037	200,600	< 0.001	50,004	199,633	< 0.001
	Female	335,785	95,804	239,981		97,410	238,375	
Urbanization	Urban	322,728	81,661	241,067	< 0.001	82,868	239,860	< 0.001
	Suburban	186,643	45,441	141,202		46,313	140,330	
	Rural	76,051	17,739	58,312		18,233	57,818	
Low income status (yes)		4842	1372	3470		1427	3415	
Charlson comorbidity index	0	557,853	136,568	421,285	< 0.001	139,901	417,952	< 0.001
	1	19,094	5699	13,395		5158	13,936	
	> = 2	8475	2574	5901		2355	6120	
Time interval (days)	mean (SD)	340.09 (545.05)	330.32 (527.81)	343.30 (550.56)	< 0.001	337.23 (531.99)	341.05 (549.37)	0.0199
	median (IQR)	77 (28–393)	82 (28–385)	77 (28–399)		84 (29–399)	77 (28–392)	
Time interval (by subgrouping, months)	< 3	259,919	63,471	196,448	< 0.001	63,426	196,493	< 0.001
	3–6	108,977	27,621	81,356		28,004	80,973	
	6–12	62,267	16,206	46,061		16,890	45,377	
	> 12	154,259	37,543	116,716		39,094	115,165	

Chi-squared tests and t-tests were used for categorical and continuous variables, respectively

*SD* standard deviation, *IQR* interquartile range

Table 2 presents the estimates for the negative binominal model with an interaction term for first-eye surgery and the time interval between the first- and second-eye surgeries. Because estimates for nonlinear models with interaction terms cannot be interpreted directly, the marginal effects were calculated for each variable (Table 3).

**Table 2:** Negative binominal model for the number of mental health consultations

	IRR	95% CI	*p*-value
First eye pre-surgery	reference		
First eye post-surgery	0.978	0.971–0.985	< 0.001
Time interval between first and second eye			
< 3 months	ref		
3–6 months	1.021	1.002–1.041	0.027
6–12 months	1.031	1.008–1.055	0.009
> 12 months	0.939	0.923–0.955	< 0.001
Post-surgery*time interval			
Post/3-6 months	1.034	1.020–1.047	< 0.001
Post/6–12 months	1.087	1.070–1.104	< 0.001
Post/> 12 months	1.095	1.082–1.108	< 0.001
Age			
20–35	ref		
35–50	1.595	1.211–2.099	0.001
50–65	2.383	1.820–3.120	< 0.001
> =65	2.870	2.192–3.756	< 0.001
Sex			
Male	ref		
Female	1.459	1.440–1.479	< 0.001
Urbanization			
Urban	ref		
Suburban	0.924	0.911–0.937	< 0.001
Rural	0.787	0.771–0.803	< 0.001
Low-income status			
No	ref		
Yes	1.738	1.602–1.885	< 0.001
Charlson Comorbidity Index			
0	ref		
1	1.221	1.180–1.264	< 0.001
> =2	1.330	1.265–1.398	< 0.001

*CI* confidence interval, *IRR* incidence rate ratio

**Table 3:** Marginal effects of first-eye surgery on the number of mental health consultations at various time intervals, full model

	Margin	95% CI	*p*-value
< 3 months	reference		
3–6 months			
Pre-surgery	0.03819	0.004–0.072	0.028
Post-surgery	0.0972	0.063–0.131	< 0.001
6 months-1 year			
Pre-surgery	0.05539	0.013–0.097	0.010
Post-surgery	0.21026	0.168–0.253	< 0.001
> 1 year			
Pre-surgery	−0.1087	−0.138--0.079	< 0.001
Post-surgery	0.04891	0.019–0.079	0.001

*CI* confidence interval

Table 3 presents the marginal effects of the first-eye surgery on the number of mental health consultations at various time intervals. The marginal effect is based on the full derivative of first-eye surgery in the negative binominal model. The results revealed that compared with the number of presurgery mental health consultations, that of postsurgery mental health consultations was reduced by a smaller magnitude for time intervals of > 3 months.

**Fig. 1:**
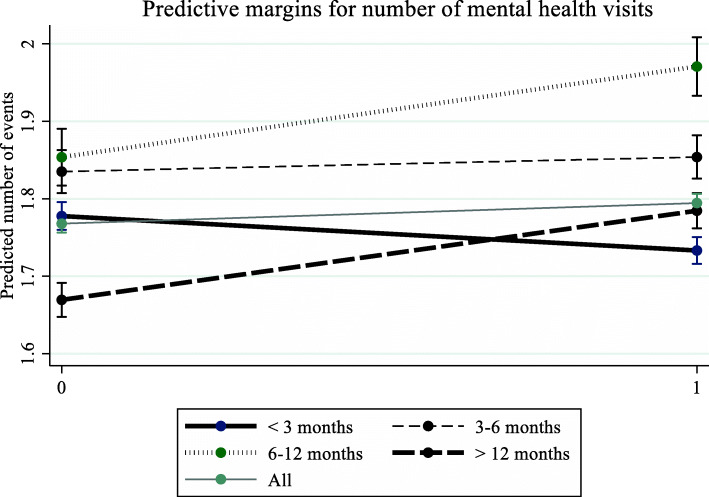
Predictive margins for number of mental health visits. Patients who underwent surgeries for both eyes were grouped into the following categories according to the time interval between surgeries: < 3, 3 to 6, 6 to 12, and > 12 months. The figure illustrates the marginal effect of first-eye surgery on the number of mental health consultations (1 year) with consideration of the time interval between first- and second-eye surgeries

Figure 1 displays the number of mental health consultations predicted by the negative binominal model with interaction effects. The overall trend revealed a slight increase in the total average number of mental health consultations after the first surgery (from 1.767 to 1.797, *p* < 0.001). However, the effect varied by time interval. The number of mental health consultations was reduced for patients with time intervals of < 3 months (1.783 to 1.743*, P* < .001), and there was a negative dose response effect such that a longer time interval corresponded to a lower reduction in the number of mental health consultations. For patients with time intervals of > 12 months, the predicted number of mental health consultations increased from 1.674 to 1.796 (*P* < .001).

## Discussion

In this study, we found that the number of mental health care consultations decreased after first-eye cataract surgery in patients who had already sought mental treatment before surgery in unadjusted analysis. We also found that the effect of decreasing the number of mental health care consultations was less pronounced in patients who had not yet received second eye cataract surgery and those who received their second eye cataract surgery more than 3 months after first eye surgery.

This study makes the following 2 contributions to the literature. First, previous studies investigating the relationship between cataract surgery and mental health topics such as depression and anxiety have produced conflicting results. Our study provides further evidence using an accurate measure of mental health consultations that first-eye cataract surgery improves patients’ mental health. Second, we investigated whether first-eye surgery helps to reduce the number of mental health consultations and whether this effect varies according to the time interval between first- and second-eye surgeries. This has not been examined in previous studies. We found that although first-eye surgery led to a lower number of mental health consultations for certain patients, this effect was observed only for those patients who also received second eye cataract surgery within 3 months of the first-eye surgery.

It should be noted that the outcome variable of this study was mental health care utilization rather than diagnoses of mental illnesses that may or may not be treatable [22]. The patients with mental health consultations identified in this study were thus patients who received treatment. However, the use of mental health consultations as an objective measure has several advantages. First, it reflects an accurate measure of the burden to society in terms of medical costs. Second, it is less prone to short-term fluctuations, which are more likely when mental health is measured only at a specific time point. Third, mental health care utilization can reflect the mental health burden for people with mental illnesses.

Given the universal health coverage under Taiwan’s NHI, our results provide valuable information for countries with similar NHI coverage rates. Because of the UHC in Taiwan, cataract surgeries are affordable and have short waiting times, and time intervals are determined more by demand-side decisions (together with physicians’ recommendations made on the basis of clinical needs) than by characteristics of the health care system. Under such a system, the time interval between the surgeries of the 2 eyes is a modifiable factor. In addition, analysis under a single-payer UHC system enabled us to eliminate supply-side uncertainties and variations and thus makes our results more robust with regard to unmeasurable supply-side confounders. The present study also expands on existing research by using a whole-population data set that allows a sufficient number of study participants to be included. Most other studies on the effects of cataract surgery and subsequent nonvisual health conditions have had small sample sizes or used non–population-based data [13, 16]. The use of such non–population-based data or data from hospital-based patients is likely to overrepresent the number of patients under specific health insurance policies and thereby influence the results by introducing possible patient selection bias and unmeasurable demand-side or supply-side confounding factors.

There are several confounding factors that warrant consideration. One may argue that longer surgical intervals can be attributed to lower-severity cataracts in the second eye. However, our results suggest that even in the case of a clinically less-severe cataract for which surgery can be delayed for more than 3 months, such a cataract can still reduce the benefit of first-eye cataract surgery with regard to the amount of mental health care utilization. Other confounders that we did not adjust for included unmeasured patient characteristics in different surgical time interval groups. Patients receiving 2 eye cataract surgeries within 3 months may be more active in seeking medical help than patients with longer intervals. In our results, mental health care utilization was most reduced in the group with time intervals of < 3 months, suggesting that for patients more active in seeking medical help, the benefits of cataract surgery for reducing mental health care consultations are notable. A plausible explanation for our results is that the effects of the surgeries for both eyes are compounded when surgeries are received within a short time interval. For patients with longer time intervals, benefits may be obtained only from surgery on the first eye. Thus, patients with longer time intervals continue to have various degrees of visual dysfunction resulting from the unoperated cataract of the second eye. Moreover, because the second-eye surgery must follow the first-eye surgery, testing the “pure” effect for each eye is impossible.

In our study, all patients received cataract surgeries for both eyes, and thus the medical costs directly related to cataract surgery should be the same, regardless of the time interval. However, medical costs indirectly related to cataract surgery (such as mental health consultations) may differ. Because of the Taiwan NHI administration’s high degree of price control, changes in per unit price due to medical inflation are unlikely over the study period. Cataracts not only directly affect vision but also indirectly affect several performance measures and have other health consequences. Walker and Lord studied 105 individuals (mean age = 73.7 years) with cataracts requiring surgery and discovered that recreational activities, reading, fine work, and daily living activities and driving behaviors accounted for the majority of visual disabilities [23]. It has also been demonstrated that cataract surgery ameliorates cognitive impairments in elderly patients [8]. Thus, finding the most effective approaches to cataract surgery for improving other nonvisual health outcomes is essential for effective medical care resource allocation.

The limitations of this study should be considered. First, increases in the number of mental health consultations are not necessarily equivalent to a greater severity of clinical mental illness. Although considering the actual number of mental health service consultations has merits, future studies should analyze the frequency of mental health consultations in combination with the actual mental health severity on a single study population. Second, our study included only patients with treated mental health conditions; since we used health insurance claims data, if a person has mental health conditions but did not make any mental health consultation, he/she would be coded as having zero mental health consultation in our dataset. The true societal mental health burden thus may have been underestimated.

## Conclusion

Cataract surgery can be resource consuming given the number of surgeries performed each year. Thus, identifying the most effective approaches to scheduling such surgeries to improve not only visual but also nonvisual outcomes is essential. For patients who are expected to undergo surgeries for both eyes within 1 year, performing both surgeries within a short time interval may be beneficial for maximizing the effect of cataract surgeries in reducing the number of mental health consultations.

## Data Availability

The dataset used in this study is available from the Statistical Office, Ministry of Health, Taiwan.

## Abbreviations

NHI: National Health Insurance
RCT: Randomized control trials
ICD-9-CM: International Classification of Diseases, 9th Revision, Clinical Modification
AIC: Akaike information criterion
